# Tips and Traps: Lessons From Codesigning a Clinician E-Monitoring Tool for Computerized Cognitive Behavioral Therapy

**DOI:** 10.2196/mental.5878

**Published:** 2017-01-11

**Authors:** Frederick Sundram, Susan J Hawken, Karolina Stasiak, Mathijs FG Lucassen, Theresa Fleming, Matthew Shepherd, Andrea Greenwood, Raechel Osborne, Sally N Merry

**Affiliations:** ^1^ Department of Psychological Medicine Faculty of Medical and Health Sciences The University of Auckland Auckland New Zealand; ^2^ Department of General Practice and Primary Health Care The University of Auckland Auckland New Zealand; ^3^ School of Health, Wellbeing and Social Care The Open University Milton Keynes United Kingdom; ^4^ Department of Paediatrics Child and Youth Health The University of Auckland Auckland New Zealand; ^5^ School of Counselling, Human Services and Social Work The University of Auckland Auckland New Zealand; ^6^ Department of Clinical Psychology The University of Auckland Auckland New Zealand; ^7^ Kapiti Youth Support Paraparaumu New Zealand

**Keywords:** e-therapy, psychotherapy, cognitive therapy, depression, psychology, adolescent, primary health care

## Abstract

**Background:**

Computerized cognitive behavioral therapy (cCBT) is an acceptable and promising treatment modality for adolescents with mild-to-moderate depression. Many cCBT programs are standalone packages with no way for clinicians to monitor progress or outcomes. We sought to develop an electronic monitoring (e-monitoring) tool in consultation with clinicians and adolescents to allow clinicians to monitor mood, risk, and treatment adherence of adolescents completing a cCBT program called SPARX (Smart, Positive, Active, Realistic, X-factor thoughts).

**Objective:**

The objectives of our study were as follows: (1) assess clinicians’ and adolescents’ views on using an e-monitoring tool and to use this information to help shape the development of the tool and (2) assess clinician experiences with a fully developed version of the tool that was implemented in their clinical service.

**Methods:**

A descriptive qualitative study using semistructured focus groups was conducted in New Zealand. In total, 7 focus groups included clinicians (n=50) who worked in primary care, and 3 separate groups included adolescents (n=29). Clinicians were general practitioners (GPs), school guidance counselors, clinical psychologists, youth workers, and nurses. Adolescents were recruited from health services and a high school. Focus groups were run to enable feedback at 3 phases that corresponded to the consultation, development, and postimplementation stages. Thematic analysis was applied to transcribed responses.

**Results:**

Focus groups during the consultation and development phases revealed the need for a simple e-monitoring registration process with guides for end users. Common concerns were raised in relation to clinical burden, monitoring risk (and effects on the therapeutic relationship), alongside confidentiality or privacy and technical considerations. Adolescents did not want to use their social media login credentials for e-monitoring, as they valued their privacy. However, adolescents did want information on seeking help and personalized monitoring and communication arrangements. Postimplementation, clinicians who had used the tool in practice revealed no adverse impact on the therapeutic relationship, and adolescents were not concerned about being e-monitored. Clinicians did need additional time to monitor adolescents, and the e-monitoring tool was used in a different way than was originally anticipated. Also, it was suggested that the registration process could be further streamlined and integrated with existing clinical data management systems, and the use of clinician alerts could be expanded beyond the scope of simply flagging adolescents of concern.

**Conclusions:**

An e-monitoring tool was developed in consultation with clinicians and adolescents. However, the study revealed the complexity of implementing the tool in clinical practice. Of salience were privacy, parallel monitoring systems, integration with existing electronic medical record systems, customization of the e-monitor, and preagreed monitoring arrangements between clinicians and adolescents.

## Introduction

Depression in adolescence is a major cause of disability that is often underreported, unrecognized [1,2], and not addressed by timely and effective treatments [3-5]. Psychological therapies are recommended as a first-line treatment for mild-to-moderate forms of depression [6,7] but are difficult to access. Computer-delivered therapies, especially those based on cognitive behavioral therapy (CBT), have been developed to help address this [8]. Computerized cognitive behavioral therapy (cCBT) can be offered as pure self-help or guided by a clinician [9,10]. The advantages of pure self-help tools include improved cost-effectiveness and nonreliance on clinicians to guide or support adolescents, and ultimately it may be some adolescents’ preference to utilize programs independently. However, there is some evidence that clinician-guided interventions or blended therapies (where face-to-face therapy and computerized approaches are used side by side) have better completion rates and are more effective than unsupported self-help therapy [11-15]. An example of a supported program is the virtual MindSpot Clinic in Australia that allows remote screening assessments (phone- or Web-based) and clinician-guided treatment for anxiety and depression [16]. On the contrary, “Beating the Blues” is an unguided Web-based depression treatment program completed by a user at home that enables the prescribing general practitioner (GP) to receive risk alerts and progress updates [17,18].

To date most cCBT programs have been designed for adults, although more recently a number of programs have been created for depressed or anxious children and adolescents. They too range from self-help tools to supported programs. For example, “Think, Feel, Do” [19] relies on a facilitator (who is not required to be a clinician) to be available during the entire delivery of the program to discuss the content covered. BRAVE-ONLINE is a clinician supported anxiety program in which young people receive weekly emails from a clinician designed to give them feedback and encourage their program progression [20]. However, BRAVE-ONLINE does not include the ability to monitor symptoms. SPARX (Smart, Positive, Active, Realistic, X-factor thoughts) is a gamified cCBT program for adolescents, which was shown to be effective in 12- to 19-year-olds presenting to primary care with mild-to-moderate symptoms of depression [21]. SPARX was designed as a pure self-help program in response to young people wanting to maintain privacy and access the program independently. A freely available self-help resource such as SPARX, which is accessible to all young people in New Zealand, can help bring treatment to the estimated three quarters of young people with depression who do not seek help [22]. In the original trial of SPARX, the referring clinicians provided a minimal degree of oversight with only brief contact after a month to ensure adequate progress. Our experience from the trial suggested that while most clinicians endorsed the use of a self-help program, some wanted to have a more formal means of monitoring adolescent users’ progress while using SPARX (Merry, personal communication).

Therefore, our study aimed to develop and implement an electronic monitoring (e-monitoring) tool relevant to adolescents with depression who attend youth-oriented primary care settings. The monitoring tool was envisaged to work in settings such as school health and counseling services because this is where many adolescents are likely to seek help [23]. To achieve this, we (1) assessed clinicians’ and adolescents’ views on using an e-monitoring tool and used this information to help shape development of the tool and (2) assessed clinician experiences with a fully developed version of the tool that was implemented in their clinical service. Of note, the tool was not expected to replace face-to-face contact but rather to offer another means of clinician oversight.

## Methods

### Study Design and Setting

A qualitative study was undertaken in New Zealand using focus groups with adolescents (aged 12-19 years) who were in contact with mental health services and also clinicians in primary care settings from the services where they worked (Table 1). We approached local secondary schools, a youth health center, and a nongovernmental organization using a snowball approach to recruit English-speaking participants. The clinicians approached were known to the researchers, and had an interest in adolescent health and in using the tool. The clinicians were GPs and allied health staff (school guidance counselors, clinical psychologists, youth workers, and nurses).

We set out to embed within SPARX an e-monitoring tool that would allow a clinician to “prescribe” SPARX and remotely oversee users’ progress and mood scores. As a minimum, the tool would have the following features:

1. A registration system for clinicians so that they could use the SPARX e-monitoring tool

2. Electronic linking of SPARX user data to the prescribing clinician

3. A dashboard accessible on the Internet to allow clinicians to track all users they had referred to SPARX

4. An algorithm to automatically generate alerts at specific and concerning levels of depression, or self-harm, and a system to encourage users to access more help

5. A system to deliver alerts or “flags” via email to the clinician

In order to track mood in SPARX, we aimed to use the Patient Health Questionnaire for Adolescents (PHQ-A) [24,25] at 3 time points: module 1 (baseline), module 4 (mid-point), and module 7 (end of intervention). We also consulted a software developer to investigate potential solutions, and schematic blueprints (wireframes) were then developed to visually guide participants through the flow of a given system and illustrate what an e-monitoring system or dashboard might look like. These were then presented at focus groups with the clinicians and adolescents, who provided feedback during the various phases of development of the tool.

### Ethics Approval and Consent

Ethics approval was obtained from the Health and Disability Ethics Committee, New Zealand (Reference: 12/CEN/62), and written informed consent was gained from each clinician, adolescent, and parents of any adolescent aged less than 16 years.

**Table 1:** Details of focus group participants during various phases of the study.

Group	Location	Focus group date	Focus group duration (minutes)	Participants	Phase
**Clinician focus groups**					
1	Youth health center	December 2012	90	3 General pratitioners (GPs) 4 Allied health staff	Phase 1 Consultation: Gauge needs and wants of clinicians and seek early feedback
2	Youth health center	December 2012	50	5 Allied health staff	
3	Primary care service	March 2013	80	11 GPs	
4	School guidance service	June 2013	60	1 GP 6 Allied health staff 3 Allied health trainees	Phase 2 Development: E-monitoring tool was beta-tested
5	School guidance service	July 2013	70	5 Allied health staff	
6	Youth health center	Dec 2013	60	3 GPs 4 Allied health staff	Phase 3 Postimplementation: Obtain postimplementation clinician feedback
7	Youth health center	Dec 2013	60	5 Allied health staff	
**Focus groups with adolescents**					
8	Youth health center	Dec 2012	90	14 Adolescents (past service users and youth advisors to the health center)	Phase 1 Consultation: Gauge needs and wants of adolescents and seek early feedback
9	Secondary school	Mar 2013	60	10 Adolescents (students who are nonservice users)	
10	Nongovernmental organization (mental health provider)	Mar 2013	70	5 Adolescents (service users)	

### Focus Groups and Data Collection

Focus groups (Table 1) were conducted during 3 phases. Phase 1 was the consultation stage and was carried out prior to the creation of the e-monitor to canvass ideas that would help the design of the e-monitoring system, based on early wireframe designs (December 2012 to March 2013). Phase 2 was the development stage and was conducted after preliminary wireframes had been revised, and it included a broader discussion of how e-monitoring could be used clinically (June to July 2013). Phase 3 was the postimplementation stage and was conducted after clinicians trialed the e-monitoring tool in day-to-day practice (August 2013 to February 2014). A semistructured schedule was used for all the focus groups (Multimedia Appendix 1), and these were digitally recorded and professionally transcribed. Each group was run by 2 of 6 experienced facilitators (FS, KS, ML, TF, MS, and SM) and the duration, while ultimately determined by the participants, was usually 60-90 minutes. FS checked fidelity of the Phases 1 and 2 clinician transcripts, and AG reviewed the transcripts of the adolescent groups and Phase 3 clinician groups.

### Data Analysis

A theoretical thematic analysis framework was used for examining the data from focus groups [26]. Transcripts were imported into NVivo before thematic analysis [27], and more specifically, the general inductive approach [28] was used to organize the dataset into multiple coded blocks. A higher level of data interpretation was then performed, and responses were assessed for what might be implied or inferred. FS checked and coded the transcripts of the Phases 1 and 2 clinician focus groups, and AG similarly reviewed and coded the transcripts of the adolescent focus groups and Phase 3 clinician focus groups. Each dataset was then independently coded (SH, Phases 1 and 2 clinician groups; MS, all adolescent groups; KS, Phase 3 clinician groups). The independent coding by the 2 researchers was then discussed until consensus was reached to determine the final themes. Quotations from focus groups, where relevant, are provided verbatim.

## Results

Common themes were identified from both the clinician and adolescent focus groups, as well as from issues reported upon as unique to either group. During Phases 1 and 2, themes were organized into 3 broad categories: clinical progress; confidentiality and privacy; and technical issues. Clinicians who were involved in clinical testing (Phase 3) reflected on some concepts (for instance, the initial themes) and drew on their “hands-on” experiences of using the actual tool postimplementation. Refinements to improve the tool were also suggested (Textboxes 1 and 2).

### Development of the E-Monitor (Phases 1 and 2)

#### Clinical Progress

##### Engagement, Adherence, and Offering Help

Most participants were positive and supportive of the idea of adding e-monitoring to enhance the effectiveness of cCBT. Some adolescents noted that it might be hard to initiate contact with a clinician if they received a message from SPARX saying “you should seek help,” and that the e-monitoring system would help clinicians to make contact with adolescents.

I think the monitoring is definitely quite key for like if you’re you know making sure you get like consistently doing it and stuff. It could be quite easier if no one was checking up on you and stuff to be like just oh that’s enough that kind of thing.

Adolescent

##### Clinical Burden and Effects on Therapeutic Relationship

E-monitoring was seen as a positive step by clinicians and adolescents, but there was also concern that it could potentially contribute to increased clinician burden. Some clinicians felt that the clinician-user therapeutic relationship may be impacted upon by a tool used in place of an ongoing face-to-face relationship, while others were concerned at the possible risk of rapid change in depression severity in adolescents. Similarly, some adolescents were concerned that clinicians might become over-burdened with the monitoring, with potential negative impacts on the clinical relationship and rapport. Clinicians and adolescents suggested the need for a discussion around e-monitoring, so that the adolescent was aware that a clinician had oversight of their progress, and could maintain the therapeutic relationship while simultaneously enhancing the adolescent’s autonomy and sense of control.

I personally think this is great, so you can give the child a bit more power if you like, to take control of how they think and what they’re going to do, because it seems often that it’s one of the parents driving what’s going to happen next and how quickly they are able to get better.

Clinician

##### Responsibility of Monitoring Risk, Parallel Monitoring, and Backup Systems

Clinicians were concerned about how they could discharge the clinical responsibility of monitoring an adolescent who was using a self-help therapy and might not be seeing the clinician regularly. In particular, there was concern about managing self-harm or suicide if the clinician was unable to monitor progress regularly due to after-hours cover, being part-time, or on annual leave. A potential solution proposed was for other people in the service to check alerts. Having a robust system in place with cross-cover arrangements would allow monitoring to continue in their absence. Similarly, having e-monitoring alerts sent to more than one clinician would allow more staff to better assess and manage risk.

I mean if I was working here and I saw it come through and I glance at it out of interest because I’m seeing the patient, I would be very happy and comfortable that the nurses had full ability to follow-up on that and someone else was doing it. It would be great. It would take too much time so that would be good.

Clinician

Adolescents realized that mood monitoring was based on honest responses by the user of the program, which may not always happen. They suggested that there should be “check-ins” alongside SPARX to ensure that the adolescent user was truly getting benefits from the treatment.

#### Confidentiality and Privacy

In keeping with the literature [29], confidentiality was an important consideration for both adolescents and clinicians. There were concerns about privacy, information use, and who had access to the information collected during the process of e-monitoring. Clinicians also had the impression that adolescents would prefer if their parents were not involved and that adolescents preferred to retain anonymity.

There was some discussion about social media, and this was in the context of adolescents possibly using their Facebook login credentials to access the cCBT program, an idea posed as a possible means of reducing registration overheads. Another idea raised was whether mental health programs should have a social media presence, given the potential stigma associated with mental illness. Adolescents were worried that others could see that they had signed up for SPARX through a Facebook announcement, and consequently asked that the option to register using a social media account be removed.

It’s like when you are depressed or something, you don’t like want to tell the whole world about it really.

Adolescent

**Key points arising during each phase.**

**Phases 1 and 2: Clinician and adolescent feedback**

*Clinicians and adolescents*

Monitoring encourages:

- adolescents to continue with therapy
- clinicians to see progress
- clinicians to offer help

Concerns:

- Clinician burden: checking email inboxes regularly for clinician alerts would interfere with clinical time
- Privacy: how information would be used and who would have access to the information
- Linking with social media: others could see whether someone was accessing computerized cognitive behavioral therapy (cCBT)
- Registration process time-consuming
- Adolescents can change their contact numbers frequently; phone/text messaging them can be difficult

Suggestions:

Autonomy in choosing what information to share and how adolescents would be contacted

 

*Clinicians only*

Concerns:

- Impaired therapeutic relationship, perceived “brush off”
- Increased clinical responsibility and managing risk
- Depression severity can change quickly and unpredictably
- Clinicians becoming the main contact point during crises
- Receiving notifications when away from work, and need to check alerts several times daily
- Alerts being sent to only one clinician
- Seeking parental involvement for adolescents viewed as an obstacle
- Amount of detail and information recorded (during registration and while completing the program) may be considered intrusive
- Cannot provide e-monitoring to populations in socially disadvantaged areas who frequently do not have reliable Internet access
- Adolescents less likely to use/check email
- Security of email and whether alerts should be sent by email
- Supportive and positive material on cCBT program may result in reduced face-to-face contact
- Not all clinicians are technically minded

Potential benefits:

- Enhance adolescent’s sense of control
- E-monitoring through the use of embedded mood screening instruments would help provide finer detail and pattern of change that is sometimes not possible with face-to-face monitoring

Suggestions:

Care needed with nonclinical staff having access to computerized records, and e-monitoring to be confined to those clinicians seen to have the necessary clinical skills

 

*Adolescents only*

Concerns:

- Challenge of initiating first contact with clinician
- Underlying motivation or honesty in answering questions to access help
- The idea of registering via Facebook credentials was rejected (even though it would be quicker or easier) due to privacy concerns

Suggestions:

- Parallel check-ins for adolescents with clinicians when completing cCBT
- Social media sites could act as a platform to improve awareness of cCBT or even normalize its use
- Clinicians could register on their behalf for e-monitoring and send adolescents the registration details
- Need for a sensitive scale to judge severity and determine urgency of help rather than yes or no answers via questionnaires
- Incentives for achieving milestones when using cCBT rather than nagging reminders on the lack of completion
- Choice of communication medium, frequency of alerts, and personalized emails rather than having generic messages
- Details on how to access more help should be provided

**Phase 3: Clinicians postimplementation**

Benefits:

- Helpful to check whether cCBT completed
- E-monitor provides alternative form of communication

Concerns:

- Increased clinical burden and extra time required to check alerts
- Privacy concerns remained

Suggestions:

- Clarify purpose of e-monitor with adolescents and what information could be accessed via the monitor
- E-monitor and alerts should be integrated with existing electronic data management systems
- Emails to alert clinicians of at-risk adolescents and to remind clinicians to log in to the e-monitoring dashboard
- Emails to provide weekly or regular summaries in terms of adolescents’ engagement and progress with SPARX (Smart, Positive, Active, Realistic, X-factor)

Reflections:

- Quality of therapeutic relationship remained good contrary to earlier expectations and, in some cases, was enhanced
- Triage person monitored alerts and then subsequently informed relevant clinicians

**Proposed solutions to issues identified.**

Before commencing e-monitoring:

- Provide support and training for clinicians on how to use the computerized cognitive behavioral therapy (cCBT) and e-monitoring systems
- Need for monitoring is dependent on the situation
- Explaining to adolescents when it is helpful to use cCBT or e-monitoring, and how and when help should be accessed
- For those who prefer not to be e-monitored, they should be able to opt out, but the cCBT program should direct adolescents to seek help
- Social media not favored as a way of registering for cCBT or e-monitoring but a way to heighten awareness of cCBT options and availability
- Clinicians who use the e-monitor should be registered with a professional regulatory body
- Only clinical staff involved in care should have access to e-monitoring data

Monitoring arrangements:

Collaboration on the details shared: provide an information sheet to adolescents and parents (if adolescents agree) clarifying monitoring arrangements or level of detail gathered, and adolescents can choose the details that will be shared via e-monitoring

- Pair with a unique clinician registration code(s)
- Registration for both the adolescent and clinician should not be time-consuming or laborious
- Completion of registration for e-monitoring together with their clinician
- Clinicians should schedule periodic face-to-face check-ins alongside cCBT
- A zero-risk approach for clinicians prescribing cCBT and e-monitoring to address concerns of clinical risk and responsibility

Alerts and seeking further help:

- Secure messaging system to alert clinicians and also to prompt adolescents
- Email alerts to clinicians about adolescents’ SPARX (Smart, Positive, Active, Realistic, X-factor) progress and weekly summaries rather than only alerts to flag concerns
- Email, phone, or text alerts to be sent to adolescents rather than any one particular medium; this is an opportunity to personalize e-monitoring and with supportive messages at certain milestones
- Clinician receipt of an alert would prompt a face-to-face assessment
- Involvement of more than one clinician in order to support work patterns; checking of alerts and clinical responsibility
- Acutely suicidal adolescents should be prompted to seek help
- Emergency helpline contact numbers that adolescents can access when distressed
- Multiagency support involving primary care, schools, 24-hour helplines, and community mental health services

Some clinicians believed there would be reduced adolescent uptake should their details be identifiable via social media. However, many clinicians did not have privacy concerns especially if details were provided on the e-monitoring arrangements to the adolescent beforehand. Both adolescents and clinicians generally agreed that SPARX users should be given the option of selecting what information they wanted to share and how to be contacted.

#### Technical Considerations

Both adolescents and clinicians agreed that the registration process for e-monitoring should not be time-consuming. Ideally, clinicians would like to do it with the individual still present in their office, time permitting. Adolescents may change their contact details frequently, and therefore discussing the right communication medium is necessary. Technical aids such as information booklets with pictorial guides could be provided. Some adolescents suggested that the registration process should be “short and sweet” and there should be shortcuts, for example, a “Play Now” button that takes you straight to the program. Others said it would be easier for their clinician to complete the registration on their behalf and then send them the details.

Some clinicians wondered who should have access to the tool and whether it should be restricted to those with appropriate clinical skills and experience. They suggested that before a clinician was given access to e-monitoring (or the ability to “prescribe” SPARX), their credentials should be verified with the relevant regulatory agencies that maintain the professional registration of clinicians. A minority of clinicians thought that the positive and supportive material within the cCBT program may cause some adolescents to avoid being e-monitored. This is because mood may improve while undergoing cCBT, and consequently the adolescent may view engagement with the clinician as unnecessary. Despite concerns about the security of emails and the way alerts would be sent, overall clinicians were supportive of e-monitoring.

Many clinicians also suggested integrating the e-monitor and alerts with existing patient management systems (including electronic mailboxes that receive laboratory results). Clinicians supported the use of the Web-based tools built into the e-monitoring system for assessing mood, for example, PHQ-A, as these would potentially allow the “teasing out” of mood symptoms in greater detail so that energy, sleep, concentration, and other dimensions could be clarified. When adolescents describe low-mood symptoms, e-monitoring would potentially allow the assessment of the pattern of change over time. Clinicians felt that the acquisition of such finer detail is currently not possible with many face-to-face meetings. However, internet connectivity is key to e-monitoring, and adolescents experiencing sociodemographic deprivation may be particularly disadvantaged in this regard.

Adolescents had a number of suggestions concerning the technical aspects of e-monitoring, but perhaps unsurprisingly, they were different from those expressed by the clinicians. Adolescents wanted to select a communication method (eg, email, texting) and agree this with the clinician. Adolescents were cognizant that the severity of symptoms varies between individuals and wanted alerts to the clinician set at a threshold high enough to warrant attention. Some also wanted to include a function that would allow the users to determine the urgency of the situation. Adolescents suggested that the following information be included when informing potential SPARX users about the ways to get help: “A recommended doctor,” “What to do if you’re depressed and you got no one,” “And if you don’t trust anyone to talk to.” Information where and how to access help (including after-hours help) should be prominent, clear, and available to all users (including those who may not need it immediately). Adolescents did not wish to receive many “unnecessary” alerts (via email or text). Therefore, the alerts going back to users should be sent at milestones (eg, completion of levels) and should not be sent too frequently. Personalized messages were favored over automated ones as they would “make you want to read it more.”

### Clinician Feedback on the E-Monitoring Tool Postimplementation (Phase 3)

Following the use of the e-monitor in clinical practice, clinicians provided feedback that was often consistent with their prior responses during the consultation and development phases of the e-monitoring tool.

#### Clinical Progress, Confidentiality, and Privacy

Clinicians noted that e-monitoring was “another thing to remember,” requiring additional work, and following up with adolescents took more time than they allocated. For many, although the e-monitor was available, it was not used. During use of the tool, one primary contact was allocated to receive all the alerts in a clinic and then notify clinicians as appropriate. This process worked well but required dedicated time. The use of e-monitoring in this way was different from how it was originally conceptualized by the clinicians.

Overall, clinicians explained that e-monitoring was helpful in checking whether adolescents had completed SPARX. They also described that e-monitoring provided another form of communication between them and the adolescent. Clinicians who used the tool believed that the quality of their relationship with adolescents was unaffected by the use of e-monitoring. Some clinicians, contrary to their earlier expectations, reported that it strengthened the therapeutic relationship as the adolescent felt more supported. Clinicians reported that e-monitoring was not on adolescents’ “radar” when they were doing it. So, it did not impact the therapeutic relationship, and clinicians continued to engage and talk with adolescents as they normally would. Overall, clinicians thought that the process worked well and shared similar views in terms of privacy considerations described earlier. They clarified with adolescents the purpose of e-monitoring and what information they could access from the e-monitor.

#### Technical Considerations

Despite attempts to keep the registration process simple, the log-on process was a barrier and “could be simpler.” Clinicians suggested an e-monitoring icon that could be integrated onto their current desktop to simplify access and serve as a reminder or prompt to check adolescents’ progress. Email alerts were widely discussed, and clinicians thought that these could serve a variety of purposes, including alerting of at-risk adolescents, reminding them to log in, and providing regular summaries in terms of adolescents’ engagement and progress with SPARX.

### Final Version of the E-Monitor

**Figure 1:**
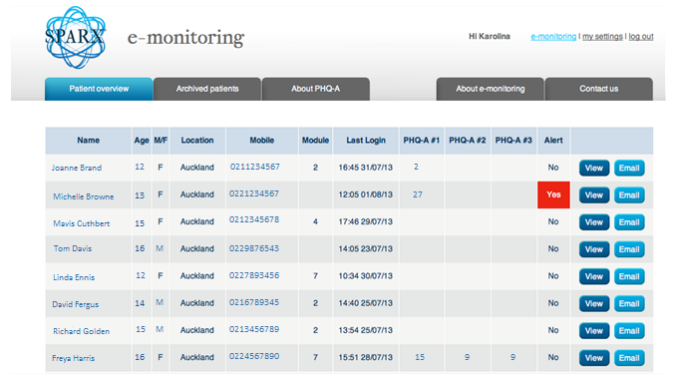
A final version of the e-monitoring dashboard following consultation with clinicians depicting fictional data of adolescents prescribed computerized cognitive behavioral therapy (cCBT) (SPARX [Smart, Positive, Active, Realistic, X-factor]), with an example of an alert. PHQ-A: Patient Health Questionnaire for Adolescents.

**Figure 2:**
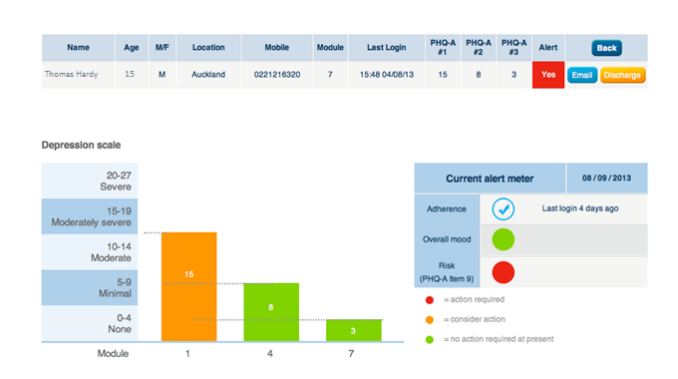
Clinician view of progress, mood, and risks for an individual prescribed computerized cognitive behavioral therapy (cCBT) (fictional data). PHQ-A: Patient Health Questionnaire for Adolescents.

In order to minimize burden on users and increase engagement with the program, mood was tracked in SPARX at the 3 time points originally proposed. Other suggestions from adolescents and clinicians are found in Textboxes 1 and 2, alongside considerations for an e-monitoring system to work effectively. Following the development and postimplementation phases, the e-monitor created (Figures 1 and 2) had the following features in (Textbox 3)

**Key features of the final version of the e-monitor.**

- A registration system for clinicians to gain access to the e-monitoring section of SPARX (Smart, Positive, Active, Realistic, X-factor), and a process to ensure that the prescribing clinician is registered with a professional regulatory body
- A unique registration code that is provided to the e-monitored adolescent, which corresponds to their prescribing clinician(s) in the service involved in their care in order to preserve privacy
- Electronic linking of adolescent user data (self-reported mood and rates of completion generated while completing SPARX) to the referring clinician
- Not linking the adolescents’ social media account to their computerized cognitive behavioral therapy (cCBT) or e-monitoring log-in
- The ability to personalize the e-monitor by the adolescent customizing the frequency of reminders and preferred communication medium with their clinician
- A Web-based dashboard to allow clinicians to track all the adolescent users (current and historical) who were prescribed SPARX
- An algorithm to automatically generate alerts at specific and concerning levels of depression or self-harm (as evidenced on a self-rated depression rating scale)
- A system to encourage adolescents to access help and contact details for emergency services
- A system of alerts sent directly to the clinician (and also weekly email updates) on the adolescent user being e-monitored

## Discussion

### Principal Findings

Our study focused on the development and use of an e-monitoring tool in clinical practice. It is therefore a timely contribution, as the area of electronically supported psychotherapy is very much in its infancy [30,31]. Using a codesign process, we developed an e-monitoring tool that would enable clinicians to electronically supervise adolescents with depression completing Web-based cCBT. There are various options for providing supported therapy, and the proposed e-monitoring tool for SPARX not only provides a mechanism for clinicians to monitor an adolescent’s progress, but also affords them an opportunity to gain regular depression assessment data via the Internet in the absence of face-to-face therapy.

In line with recent treatment preferences, clinicians were in favor of a system of monitoring adolescents with mild-to-moderate depression that embraced technology, various communication modalities, and a clinician-adolescent shared decision-making approach [32,33]. E-monitoring in tandem with face-to-face check-ins could potentially address clinician and adolescent concerns of no one providing oversight of a cCBT program such as SPARX, and this is a solution that has been previously identified in the literature [34]. While computer-based clinician support systems are increasingly gaining traction, it is also important that these do not place additional demands on the clinician’s workload [35]. Providing e-monitoring as well as face-to-face supervision may result in duplication of assessments and an increased clinician workload, but the need for such additional face-to-face assessment may reflect the reluctance of clinicians in depending on systems other than their own clinical judgment gathered from direct clinical contact.

Clinicians in our study also recognized the potential difficulties associated with after-hours monitoring and responding to concerns. Therefore, monitoring arrangements should be preagreed with adolescents in relation to checking alerts and also with regard to their preferred communication methods (phone, text alerts, emails). As has been recommended by others, relevant contact numbers can be provided by a GP [36] should a user or client experience a crisis, or these details could be embedded in the cCBT program, or alternatively, integrated between hotlines or emergency services and the cCBT program. Whereas the latter would be a novel and useful contribution (because most programs do not have such integration built-in), there may be additional challenges to implement this, as it involves a wider group of stakeholders. Additionally, clinicians would like e-monitoring integrated with the existing patient data management systems they are familiar with for receiving and monitoring alerts. Logistically this is a challenging prospect, as clinicians in primary care utilize different systems, and there may be little motivation on the part of the developers of patient data management systems to include e-monitors (like the one we have developed) into their systems.

There have been e-monitoring studies on various health conditions such as asthma and human immunodeficiency virus infection focusing on treatment adherence [37,38]. However, there are only a limited number of studies that have directly involved clinicians in a codesign process. For example, a Swiss qualitative study conducted with pharmacists on the implementation of an e-monitoring system for supporting treatment adherence similarly highlighted some of the difficulties we encountered, specifically the challenges of integrating an e-monitor into existing systems, but preexisting collaboration with physicians on codesigning the system helped [39]. Understandably clinicians would like to use an e-monitoring system that is simple to access, but also ensures privacy and confidentiality. Future developments should strive to fully integrate e-monitoring into existing data management systems, as challenging as this may be, so that e-monitoring is maximally useful to clinicians and acceptable to users [40].

During the development phase of the e-monitor in our study, clinicians noted several concerns. However, when they were followed up after using the tool postimplementation, they found that some of the initially identified concerns did not eventuate. For example, clinicians expected a negative impact on the therapeutic relationship but this did not arise. Also, during the development phase, alerts were envisaged to be sent to clinicians to highlight adolescents who might be critical in terms of mood severity or risks. However, they used the tool somewhat differently from what had been proposed during the postimplementation phase, because clinicians flagged that it required additional time for e-monitoring to occur. So, a dedicated clinician at the primary care service reviewed the dashboard and alerts regularly, and informed the other monitoring clinicians about the progress of their adolescent users. This was not how the e-monitor was originally designed to work, but it demonstrated a practical adaptation at this particular service. As clinicians are also concerned about the practicalities of e-monitoring, when a GP becomes the main crisis-intervention provider, there have been suggestions of a “zero-risk” approach for GPs and sharing of clinical responsibility with crisis teams [36,41].

Adolescents valued their privacy and preferred not to use their social networking login details. They also preferred a quick registration process, but one that either they or clinicians could undertake on their behalf. Moreover, adolescents wanted to discuss and customize e-monitoring arrangements and the timing and frequency of feedback they received via the e-monitor. While adolescents preferred their parents not to be involved in the e-monitoring process, they welcomed parallel monitoring with face-to-face check-ins with their clinician, despite reported difficulties with adolescents seeking help or expressing their concerns directly via face-to-face conversations [42]. Adolescents also wanted information on how to access support, especially after-hours when their clinician might be unavailable. As most adolescents have access to the Internet [43], this makes a Web-based e-monitoring system feasible in practice. With regard to SPARX (with or without e-monitoring), it has the capacity to advise adolescents to seek immediate help if they achieve a high score on mood symptoms or risk, but in practice, it may be difficult for the adolescent to ask for help. Information sheets could be provided prior to e-monitoring or during primary care consults, as depressed adolescents often do not recall details of consultations due to impaired concentration [36].

### Limitations

This qualitative study involved adolescents and clinicians in New Zealand who, respectively, had experienced mental health difficulties and those who had managed adolescents with depression in primary care. The main limitation was that we were unable to take our interpretations of the data back to the participants for verification, which limited the study’s trustworthiness. Additionally, we were not able to undertake a systematic evaluation of the e-monitoring tool at multiple primary care sites or involve adolescents in the postimplementation phase. This was due to time and resource constraints. However, the multidisciplinary research team consisted of experienced clinicians who were directly involved in adolescent mental health and therefore very familiar with the issues.

### Conclusions

Depression often commences during a critical period of early development, so addressing the needs of adolescents yields not only significant improvements in their health care outcomes, but also longer-term benefits over their life span [44,45]. Clinician and adolescent views were acquired on e-monitoring for cCBT, an area with little previous empirical research and even less so in primary care where adolescents are usually seen for depression. Subsequently, an e-monitoring tool was designed in line with clinicians’ wishes for oversight of adolescents using SPARX. The e-monitor was also designed with the “needs and wants” of adolescents and included a system that was easy to use with information and alerts. We are currently exploring ways in which the e-monitor for SPARX can be integrated into patient data management systems. Overall, clinical progress, confidentiality or privacy, and technical issues need to be considered alongside discussion between the clinician and the adolescent prior to commencement of e-monitoring arrangements. The findings from this study are potentially applicable beyond the adolescent population and will be of interest to developers of the various cCBT packages who may be considering a monitoring or feedback mechanism.

## Appendix

Multimedia Appendix 1 Semi-structured schedule used with focus groups.

## Abbreviations

CBT: cognitive behavioral therapy
cCBT: computerized cognitive behavioral therapy
GP: general practitioner
PHQ-A: Patient Health Questionnaire for Adolescents
SPARX: Smart, Positive, Active, Realistic, X-factor

## References

[1] Costello EJ, Erkanli A, Angold A (2006). Is there an epidemic of child or adolescent depression?. J Child Psychol Psychiatry.

[2] Lewinsohn PM, Pettit JW, Joiner Jr TE, Seeley JR (2003). The symptomatic expression of major depressive disorder in adolescents and young adults. J Abnorm Psychol.

[3] Kessler RC, Avenevoli S, Merikangas KR (2001). Mood disorders in children and adolescents: an epidemiologic perspective. Biol Psychiatry.

[4] Wilkinson P, Dubicka B, Kelvin R, Roberts C, Goodyer I (2009). Treated depression in adolescents: predictors of outcome at 28 weeks. Br J Psychiatry.

[5] Mariu KR, Merry SN, Robinson EM, Watson PD (2012). Seeking professional help for mental health problems, among New Zealand secondary school students. Clin Child Psychol Psychiatry.

[6] (2006). Computerised Cognitive Behaviour Therapy for the Treatment of Depression and Anxiety.

[7] Richardson T, Stallard P, Velleman S (2010). Computerised cognitive behavioural therapy for the prevention and treatment of depression and anxiety in children and adolescents: a systematic review. Clin Child Fam Psychol Rev.

[8] Kenardy J, Adams C (1993). Computers in cognitive-behaviour therapy. Aust Psychol.

[9] Naslund JA, Marsch LA, McHugo GJ, Bartels SJ (2015). Emerging mHealth and eHealth interventions for serious mental illness: a review of the literature. J Ment Health.

[10] Dowrick C (2015). Computerised self help for depression in primary care. Br Med J.

[11] Perini S, Titov N, Andrews G (2009). Clinician-assisted Internet-based treatment is effective for depression: randomized controlled trial. Aust N Z J Psychiatry.

[12] Høifødt RS, Lillevoll KR, Griffiths KM, Wilsgaard T, Eisemann M, Waterloo K, Kolstrup N (2013). The clinical effectiveness of web-based cognitive behavioral therapy with face-to-face therapist support for depressed primary care patients: randomized controlled trial. J Med Internet Res.

[13] van der Vaart R, Witting M, Riper H, Kooistra L, Bohlmeijer ET, van Gemert-Pijnen LJ (2014). Blending online therapy into regular face-to-face therapy for depression: content, ratio and preconditions according to patients and therapists using a Delphi study. BMC Psychiatry.

[14] Wentzel J, van der Vaart R, Bohlmeijer ET, van Gemert-Pijnen JE (2016). Mixing online and face-to-face therapy: how to benefit from blended care in mental health care. JMIR Ment Health.

[15] Kleiboer A, Donker T, Seekles W, van Straten A, Riper H, Cuijpers P (2015). A randomized controlled trial on the role of support in Internet-based problem solving therapy for depression and anxiety. Behav Res Ther.

[16] Nielssen O, Dear BF, Staples LG, Dear R, Ryan K, Purtell C, Titov N (2015). Procedures for risk management and a review of crisis referrals from the MindSpot Clinic, a national service for the remote assessment and treatment of anxiety and depression. BMC Psychiatry.

[17] Hunt S, Howells E, Stapleton B (2006). The addition of a computerised cognitive behavioural therapy program, to a stepped care, primary care mental health service. The Journal of Primary Care Mental Health.

[18] Proudfoot J, Goldberg D, Mann A, Everitt B, Marks I, Gray JA (2003). Computerized, interactive, multimedia cognitive-behavioural program for anxiety and depression in general practice. Psychol Med.

[19] Stallard P, Richardson T, Velleman S, Attwood M (2011). Computerized CBT (Think, Feel, Do) for depression and anxiety in children and adolescents: outcomes and feedback from a pilot randomized controlled trial. Behav Cogn Psychother.

[20] Spence SH, Donovan CL, March S, Gamble A, Anderson R, Prosser S, Kercher A, Kenardy J (2008). Online CBT in the treatment of child and adolescent anxiety disorders: issues in the development of BRAVE–ONLINE and Two Case Illustrations. Behav Cogn Psychother.

[21] Merry SN, Stasiak K, Shepherd M, Frampton C, Fleming T, Lucassen MF (2012). The effectiveness of SPARX, a computerised self help intervention for adolescents seeking help for depression: randomised controlled non-inferiority trial. Br Med J.

[22] Sawyer MG, Arney FM, Baghurst PA, Clark JJ, Graetz BW, Kosky RJ, Nurcombe B, Patton GC, Prior MR, Raphael B, Rey JM, Whaites LC, Zubrick SR (2001). The mental health of young people in Australia: key findings from the child and adolescent component of the national survey of mental health and well-being. Aust N Z J Psychiatry.

[23] National Research Council & Institute of Medicine (2009). Preventing mental, emotional, and behavioral disorders among young people: Progress and possibilities.

[24] Johnson JG, Harris ES, Spitzer RL, Williams JB (2002). The patient health questionnaire for adolescents: validation of an instrument for the assessment of mental disorders among adolescent primary care patients. J Adolesc Health.

[25] Spitzer RL, Kroenke K, Williams JB (1999). Validation and utility of a self-report version of PRIME-MD: the PHQ primary care study. Primary Care Evaluation of Mental Disorders. Patient Health Questionnaire. J Am Med Assoc.

[26] Tong A, Sainsbury P, Craig J (2007). Consolidated criteria for reporting qualitative research (COREQ): a 32-item checklist for interviews and focus groups. Int J Qual Health Care.

[27] Braun V, Clarke V (2006). Using thematic analysis in psychology. Qual Res Psychol.

[28] Thomas DR (2006). A general inductive approach for analyzing qualitative evaluation data. Am J Eval.

[29] Ford CA, Millstein SG, Halpern-Felsher BL, Irwin Jr CE (1997). Influence of physician confidentiality assurances on adolescents' willingness to disclose information and seek future health care. A randomized controlled trial. J Am Med Assoc.

[30] Wilhelmsen M, Høifødt RS, Kolstrup N, Waterloo K, Eisemann M, Chenhall R, Risør MB (2014). Norwegian general practitioners' perspectives on implementation of a guided web-based cognitive behavioral therapy for depression: a qualitative study. J Med Internet Res.

[31] Montero-Marín J, Prado-Abril J, Botella C, Mayoral-Cleries F, Baños R, Herrera-Mercadal P, Romero-Sanchiz P, Gili M, Castro A, Nogueira R, García-Campayo J (2015). Expectations among patients and health professionals regarding Web-based interventions for depression in primary care: a qualitative study. J Med Internet Res.

[32] Gray JAM (2002). The Resourceful Patient.

[33] Powell JA, Darvell M, Gray JAM (2003). The doctor, the patient and the world-wide web: how the internet is changing healthcare. J R Soc Med.

[34] Rhodes H, Grant S (2012). A review of computerised cognitive behavioural therapy (cCBT) for depression. Cumbria Partnership Journal of Research Practice and Learning.

[35] Miller P, Phipps M, Chatterjee S, Rajeevan N, Levin F, Frawley S, Tokuno H (2014). Exploring a clinically friendly web-based approach to clinical decision support linked to the electronic health record: design philosophy, prototype implementation, and framework for assessment. JMIR Med Inform.

[36] Falconer M, Arroll B (2012). Beating the Blues the online way. New Zealand Doctor.

[37] Chan AHY, Reddel HK, Apter A, Eakin M, Riekert K, Foster JM (2013). Adherence monitoring and e-health: how clinicians and researchers can use technology to promote inhaler adherence for asthma. J Allergy Clin Immunol Pract.

[38] Anderson 3rd WC, Szefler SJ (2015). New and future strategies to improve asthma control in children. J Allergy Clin Immunol.

[39] Marquis J, Schneider MP, Spencer B, Bugnon O, Du Pasquier S (2014). Exploring the implementation of a medication adherence programme by community pharmacists: a qualitative study. Int J Clin Pharm.

[40] De Bleser L, De Geest S, Vincke B, Ruppar T, Vanhaecke J, Dobbels F (2011). How to test electronic adherence monitoring devices for use in daily life: a conceptual framework. Comput Inform Nurs.

[41] de Montalk J (2012). 'Zero-risk' e-tool beats the blues. New Zealand Doctor.

[42] Coyle D, McGlade N, Doherty G, O'Reilly G (2011). Exploratory evaluations of a computer game supporting cognitive behavioural therapy for adolescents.

[43] Madden M, Lenhart A, Duggan M, Cortesi S, Gasser U http://www.pewinternet.org/2013/03/13/teens-and-technology-2013/.

[44] Simon GE, Ludman EJ (2009). It's time for disruptive innovation in psychotherapy. Lancet.

[45] Weller EB, Weller RA (2000). Depression in adolescents growing pains or true morbidity?. J Affect Disord.

