# Supplementary material for: Tips and Traps: Lessons From Codesigning a Clinician E-Monitoring Tool for Computerized Cognitive Behavioral Therapy
Source: JMIR Ment Health. 2017 Jan 11;4(1):e3. doi: 10.2196/mental.5878 (PMC5266827; doi:10.2196/mental.5878)
Supplement: Multimedia Appendix 1 [file mental_v4i1e3_app1.pdf]

E-monitoring focus groups – questions for clinicians

1) Explain SPARX and the proposed e-monitoring tool. Ask participants if they have used SPARX, or have used other cCBT tools, and in particular if they have referred their clients to it and what did that process look like.
2) As a clinician, do you see yourself 'prescribing' SPARX, or would you prefer letting the young person use it on their own (i.e. pure self-help). Please comment on the following implications:
   - Time spent reviewing cases;
   - Monitoring risk of self-harm;
   - Clinical responsibility;
   - Technical savviness;
   - Saving time;
   - Using the stepped care approach; and,
   - Which professional groups may find it most useful?
3) Who should have access to e-monitoring?
   - Primary clinician vs. support from other clinical staff.
     - What happens when someone is on leave?
     - Would you like to nominate a secondary email (e.g. practice nurse)?
   - What about monitoring by other non-clinical people – e.g. nominated peer support person, parents, etc.?
4) School counselors – how do you see e-monitoring fitting with your practice?
5) Regarding setting up clinicians to use e-monitoring – what steps should we take to verify/vet people? (e.g. doctors, nurses, etc. have professional registration numbers we could check but what about other professionals?)
6) What training would people like to get in using SPARX and e-monitoring? Would a web tutorial or a podcast/video cast be acceptable?
7) What specific changes would you like to 'track' or what features do you want to consider?
   - Mood?
   - Risk?
   - Adherence and when/how often adolescent uses SPARX?
   - Getting emails/alerts/requests of help from a client?
   - Being able to email and/or text clients (from within the system)?
   - Reviewing progress on the dashboard vs. getting alerts (how often would they check the dashboard, how often do they check emails)?
   - Other information from the adolescent?

Show e-monitoring wireframes
(iterative process during the development phase)

8) First impressions?
9) Have we missed out something obvious?
10) Does it look like it has covered most of the important things?

11) Does it look easy to use/navigate?
12) Do people know PHQ-A?
    - How can we make interpretation of the results easier?
      e.g. shading, legend use, color coding, mouse over?
13) If it looked like this, would people be happy to use it?
14) How often can you imagine yourself checking this dashboard?
15) Is there any information you would like to add?
16) Is there anything we need to take out (e.g. too much information, too confusing)?
17) Other comments?

E-monitoring focus groups – Questions for adolescents

1) Explain SPARX, SPARX online and the e-monitoring tool.
2) Ask how many have heard of SPARX, or other online cCBT tools or interactive websites for young people with depression (or for health related information).
    - Have you ever been given information by your doctor/counselor about things that are useful online?
    - Would you be prepared to go online to do a program like SPARX if you were down/stressed/feeling like you needed some support?
3) If your doctor/counselor suggested that you could try SPARX online – would you like to do it on your own (self-help, unsupervised) or would you like to know that your clinician can see your progress and could provide extra support if needed?
    - Discuss advantages/disadvantages of e-monitoring versus self-help use
        - Who is it most suited for?
        - When is one better than the other?
        - What about privacy?
        - Autonomy – being able to choose and being in control?
        - Knowing that someone is there to give more help?
        - Other thoughts?
4) Who should have access to e-monitoring?
    - Would you want your clinician to get emails if you said that you were not feeling well, getting worse, or felt suicidal?
    - What about nominating other people (not just clinicians) to access the e-monitoring or receive emails from the system?
        - Peer support person?
        - Parent?
        - Another adult you trust?
5) If applicable - what about e-monitoring in different situations – e.g. having it at school (with a school counselor) versus in a health clinic?
6) What information would you like/be prepared to share with your clinician/counselor via e-monitoring?
    - Mood?
    - Risk?

- When and how many modules you've done (adherence)?
- Getting (personalized) emails from your clinician?
- Getting automated emails from the system?
- Getting text messages from your clinician?
- Getting text messages from the system?
- Getting Facebook messages from the system?
- Ability to opt in/out to receive emails regarding your clients – pros/cons, why?
- Emails sent to your support person? Who? When may it be acceptable?
- Other information/suggestions from the group?

Show e-monitoring wireframes

7) Explain process of having SPARX prescribed, with a unique ID and then display wireframes.
8) First impressions?
9) Have we missed out something obvious?
10) Does it look like it's covered most of the important things?
11) Logging in with Facebook? Good idea or not?
12) Does it look easy to use/navigate?
13) Do people know PHQ-A? (explain that the results are for the clinician to see and that their graph looks different within SPARX – more youth friendly and less 'clinical').
    - How can we make interpretation of the results easier?
      e.g. shading, legend use, color coding, mouse over?
14) If it looked like this, would you be happy for clinicians to see this information?
15) Is there any information you would like to add?
16) Is there anything we need to take out (too much information, too confusing?)
17) Other comments?
18) Unique ID – would people keep it private? What's the risk of others getting hold of it and using it to log into SPARX?
